# Development of Attention-based Prediction Models for All-cause Mortality, Home Care Need, and Nursing Home Admission in Ageing Adults in Spain Using Longitudinal Electronic Health Record Data

**DOI:** 10.1007/s10916-024-02138-z

**Published:** 2025-01-25

**Authors:** Lucía A. Carrasco-Ribelles, Margarita Cabrera-Bean, Sara Khalid, Albert Roso-Llorach, Concepción Violán

**Affiliations:** 1 Institut Universitari d’Investigació en Atenció Primària Jordi Gol (IDIAP Jordi Gol), Barcelona, Spain; 2 Department of Signal Theory and Communications, Universitat Politècnica de Catalunya (UPC), Barcelona, Spain; 3 Unitat de Suport a la Recerca Metropolitana Nord, Institut Universitari d’Investigació en Atenció Primària Jordi Gol (IDIAP Jordi Gol), C/ Mare de Déu de Guadalupe, 2, Mataró, 08303 Barcelona Spain; 4 Planetary Health Informatics Lab, University of Oxford, Oxford, UK; 5 Direcció d’Atenció Primària Metropolitana Nord, Institut Català de la Salut, Badalona, Spain; 6 Fundació Institut d’Investigació en ciències de la salut Germans Trias i Pujol (IGTP), Badalona, Spain; 7 Universitat Autònoma de Barcelona, Cerdanyola del Vallès, Spain

**Keywords:** Attention mechanism, Electronic health records, Longitudinal data, Predictive modeling, Primary care, Recurrent neural networks

## Abstract

Predicting health-related outcomes can help with proactive healthcare planning and resource management. This is especially important on the older population, an age group growing in the coming decades. Considering longitudinal rather than cross-sectional information from primary care electronic health records (EHRs) can contribute to more informed predictions. In this work, we developed prediction models using longitudinal EHRs to inform resource allocation. In this study, we developed deep-learning-based prognostic models to predict 1-year and 5-year all-cause mortality, nursing home admission, and home care need in people over 65 years old using all the longitudinal information from EHRs. The models included attention mechanisms to increase their transparency. EHRs were drawn from SIDIAP (primary care, Catalonia (Spain)) from 2010-2019. Performance on the test set was compared to that from baseline models using cross-sectional one-year history only. Data from 1,456,052 individuals over 65 years old were considered. Cohen’s kappa obtained using longitudinal data was 3.4-fold (1-year all-cause mortality), 10.3-fold (5-year all-cause mortality), 1.1-fold (5-year nursing home admission), and 1.2-fold (5-year home care need) higher than that obtained by the one-year history baseline models. Our models performed better than those not considering longitudinal data, especially when predicting further into the future. However, nursing home admission and home care need in the long term were harder to predict, suggesting their dependence on more abrupt changes. The attention maps helped to understand the predictions, enhancing model transparency. These prediction models can contribute to improve resource allocation in the general population of aging adults.

**Supplementary Information:**

The online version contains supplementary material available at 10.1007/s10916-024-02138-z.

## Introduction

A higher life expectancy together with the decline in birth rates is leading to the ageing of the global population [1, 2]. For instance, the percentage of people over 65 years of age in Spain is expected to grow from 19.6% at present to 34.4% by 2050 [3]. In this context, resource management, healthcare strategies, and policy-making need to be reconsidered to maintain healthcare systems. The prediction of health-related outcomes in the general population over 65 years of age, such as healthcare services utilization and mortality could help improving resource planning.

Developing an accurate predictive model for the general population can be challenging. Having as much information as possible, both in terms of variables and patients, is advisable to enhance their generalisability and capture diverse scenarios. Models based on Artificial Intelligence (AI) are generally capable of handling larger numbers of variables than classical statistical models with which most clinical risk scores have been developed [4, 5]. The rise in the use of electronic health records (EHRs), representing a longitudinal and comprehensive description of the patient’s health history, has increased the number of large-scale longitudinal studies, such as those targeting the general population or developing AI models. Working with EHRs can be challenging, since the records are usually heterogeneous (in terms of quantity and data domains registered), irregularly sampled, and riddled with mistakes and missing data [6]. These characteristics are typical of real-world, i.e. routinely collected, data [7].

AI-based models are traditionally considered “black boxes” since knowing which variables are most determinant for prediction is not as immediate as with statistical models. However, the last decade has seen substantial efforts to overcome this drawback through the development of methods such as attention mechanisms [8–10] or model-agnostic techniques (e.g. LIME [11], SHAP [12]). These techniques can help to understand which clinical variables contributed the most to the outcome. Moreover, if longitudinal data are used, this contribution can be obtained both at the variable and time level [13–15].

In this work, we developed an AI-based prognostic model able to: (1) handle different types of variables, (2) deal with missing values, (3) handle longitudinal data sequentially, (4) handle participants having different lengths of history, and (5) report the contribution of each period and of each variable at each time period to the predicted outcome. The proposed architecture is based on recurrent neural networks (RNN) and incorporates hierarchical attention mechanisms at both variable and time period levels, being able to use real-world EHR data to generate predictions more transparently. We called it ARIADNEhr (Attention-based pRediction on longItudinAl Data iN Ehr). The ultimate goal of ARIADNEhr is to make predictions that can inform resource allocation in the public healthcare system.

## Methods

This study complied with the Transparent Reporting of a multivariable prediction model for Individual Prognosis or Diagnosis (TRIPOD) statement [16] (see Supplementary Material). Approval was obtained from the Ethical Committee of Fundació Institut Universitari per a la recerca a l’Atenció Primària de Salut Jordi Gol i Gurina (IDIAPJGol) (#19/518-P).

### Data Source

Data were drawn from the Information System for Performing Primary Care Research (SIDIAP) [17]. This database contains pseudo-anonymized, routinely collected health data from primary care centers in Catalonia managed by the Catalan Health Institute (CHI), and accurately represents almost 80% of the Catalan population [18]. Data available in SIDIAP include sociodemographic information, visits to primary care, clinical measures (e.g. BMI, blood pressure, questionnaires), all diagnoses made in primary care (using International Classification of Diseases, $10^{th}$ revision, ICD-10 [19]), laboratory results, medication dispensed in pharmacies (using Anatomical Therapeutic Classification, ATC, 5$^{th}$ level [20]) and inclusion in social assistance programs. The CHI can link SIDIAP data to publicly-funded hospital admissions. SIDIAP database is based on opt-out presumed consent. If a patient decides to opt-out, their routine data would be excluded from the database. Data were obtained from SIDIAP after study approval, and all authors had access to the database.

### Study Population

**Fig. 1:**
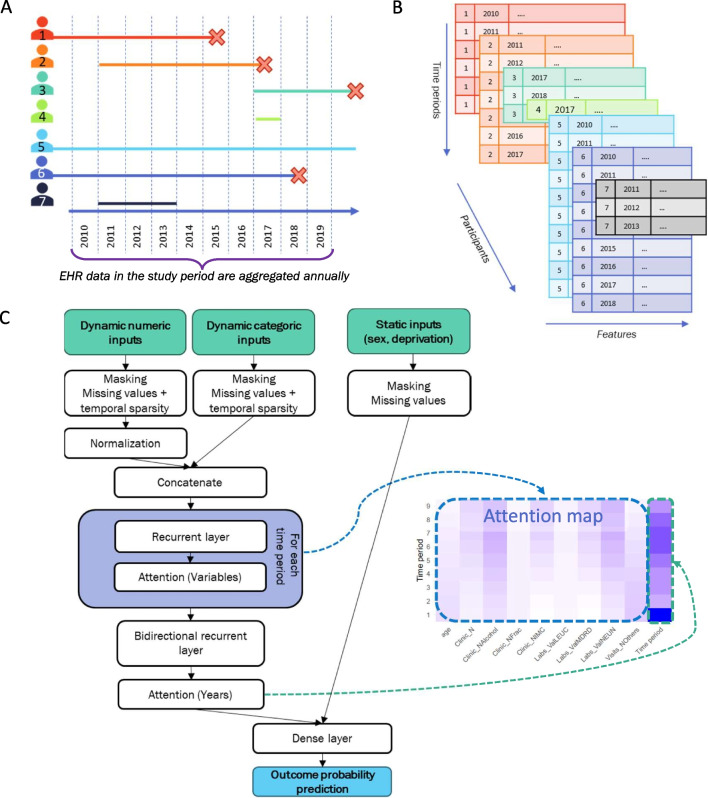
Diagrams from the study design to prediction modeling. *Note:* Figure 1A shows 7 examples of participants dynamically entering the cohort as they met the inclusion criteria. A red cross represents the outcome of interest. These participants’ EHR data are aggregated on a calendar year basis. Patient 4 would not be considered for the models, as they do not have enough follow-up data. Figure 1B shows how the aggregated EHR data are aggregated into a tensor that is fed to the model represented in Fig. 1C, which represents ARIADNEhr’s architecture. This subfigure also illustrates which part of the architecture generates each part of the attention map (on the right) that contributes to the model’s transparency

A longitudinal study was designed, involving a dynamic cohort from primary care services provided by CHI in Catalonia, Spain, from 1 January 2010 to 31 December 2019. People aged 65 years or older were included at baseline, while new patients were added as they turned 65 or arrived in the catchment area (if already aged $\ge$65 years) during the follow-up period. Follow-up continued until death, transfer out of the catchment area (loss to follow-up), or study end. Figure 1A shows a scheme of this type of study. Individuals with no available data, no visits to their primary care centre over the study period, or aged 100 years or older at the start of the study were excluded (see Fig. S1).

### Features and Outcomes

Incorrect sex-specific diagnoses or those with inconsistent dates, along with duplicate diagnoses (same person, same day, same code) were excluded. Then, as Fig. 1B shows, EHR data were aggregated annually. This involved creating 60 variables for chronic disease occurrence, as in [21]; 37 variables for frailty deficits, as in [22]; 67 variables for laboratory tests counts, mean values, and number of tests out of reference values, and 50 variables for clinical measurements counts and mean values (or mode, if the variable was categorical). Furthermore, 8 variables reported primary care consultations, 9 variables for hospital admissions, 3 variables for billed drugs, and other 12 variables for specific events. This annual aggregation was chosen over a more temporally detailed one because the data source was primary care and visiting the GP several times in a single year is uncommon, especially in younger people. Therefore, a greater temporal granularity would have increased the number of missing values. Additionally, static information on sex assigned at birth and socioeconomic status [23] was included, totalling 248 variables (listed in [24]).

The outcomes were all-cause mortality, nursing home admission, and need for home care. The last two were obtained from the diagnoses recorded in the primary care EHR: ICD-10 codes starting with Z74 meant home care, while the ICD-10 code Z59.3 signified admission to a nursing home [19].

### Attention-based Model Definition

In this work, we present ARIADNEhr (Attention-based pRediction on longItudinAl Data iN Ehr). ARIADNEhr includes recurrent layers to consider longitudinal data sequentially, two-level masking layers to work with irregularly-sampled and missing data, and a two-level attention mechanism to increase the transparency of the model (see Fig. 1C).

**Table 1:** Best hyperparameters for each outcome and task

Outcome	Prediction window	RNN type	RNN units	Dense layer before output
All-cause mortality	1 year	GRU	256	No
All-cause mortality	5 years	GRU	256	Yes
Nursing home admission	1 year	LSTM	256	No
Nursing home admission	5 years	GRU	512	No
Home care need	1 year	GRU	256	Yes
Home care need	5 years	GRU	256	No

To ensure consistent temporal dimensions among patients with varying follow-up lengths, zero-padding was applied to the input tensor (Fig. 1B). Masking was used twice: first, to indicate zero-padded periods to the model, and second, to flag missing values within each period. Masking creates a matrix of the input data dimension to indicate real EHR values. In this study, missing data, which in the case of EHRs are often unrecorded rather than recorded and missed data, was exclusively addressed through the masking layer. After masking, numerical data underwent normalization, centering the data distribution around 0 with a standard deviation of 1, before concatenating categorical data.

The variables at each time period were processed using a recurrent layer, the outputs of which were used to calculate their attention weights. These weights represent the contribution of each EHR variable at each time period to the outcome. The outputs of every time period were then concatenated and fed to a bidirectional recurrent layer, sequentially processing inputs in the time domain. Its outputs were processed by a second attention mechanism, calculating the overall contribution of each time period to the outcome. The output of this last layer per patient was concatenated to the static variables to compute the outcome probability. For the complete formulation of ARIADNEhr, please refer to the Supplementary Material.

### Model Development and Performance Evaluation

A holdout split was used to obtain the training (90%) and test set (10%). For hyperparameter tuning, the training set was again split into training (85%) and validation (15%) sets. The following hyperparameters were tuned using a grid search on these new training and validation sets, targeting ROC-AUC. *RL* type: [GRU, LSTM], *RL* units (*U*): [64, 128, 256, 512], *DL* layers: [0, 1]. The same *RL* type and units (*U*) were set for attention at variable and time levels.

After identifying the best hyperparameters (Table 1), the training and validation sets were merged for the final training of the model, and then its performance was measured on the test set. Metrics on the test set are reported with a 95% confidence interval (CI), using 1000 bootstrap samples. The following metrics were used to evaluate the performance: area under the ROC curve (ROC-AUC); recall, i.e., sensitivity; precision, and area under the precision-recall curve (PR-AUC). In addition, calibration plots were calculated to assess the agreement between predicted and observed probabilities of the outcomes in different percentiles of the predicted value. The development of the model was performed in Tensorflow and Python 3.9. Additional details on the training can be found in the Supplementary Material.

ARIADNEhr’s performance was assessed by comparing it to several simpler approaches (i.e., logistic regression, random forest, and XGBoost), which were trained using data from the first year of the study to predict outcomes for the following year or after 5 years. In cases where there were missing data during that year, imputation was carried out by using the mean or mode values of individuals of the same age and sex. These baseline models were trained on R v4.2, using the default hyperparameters as defined in the *mlr3* package [25]. For the sake of comparability, the same training and test sets that were used for training and evaluating ARIADNEhr were employed for these baseline models. Their performance is also reported with 95% confidence intervals, based on 1000 bootstrap resamples of the test dataset.

## Results

### Patient Characteristics

Of the initial sample of 1,702,062 individuals that were extracted from SIDIAP (i.e., primary care EHR database from Catalonia, Spain) [18], 1,456,052 met the inclusion criteria and were included (Fig. S1). These participants had a mean follow-up of 7.0 years (standard deviation: 3.2) in the study period (2010/01/01-2019/12/31) and 55.8% were female (see Table 2). For the development of the 1-year and 5-year models, individuals with at least 1 year of follow-up (N = 1,364,819; 93.7%) and at least 5 years of follow-up (N = 976,404; 67.1%) were selected, respectively. Their description in terms of age, sex, and socioeconomical status was very similar to that of the general population.

**Table 2:** Demographic characteristics of the total study population and of those individuals used to develop the 1-year and 5-year prediction models

Variable	Prediction 1-year ahead N = (1,364,819, 93.7%)		Prediction 5-years ahead N = (976,404, 67.1%)		Total (N = 1,456,052)
	Train (N = 1,228,337)	Test (N = 136,482)	Train (N = 878,763)	Test (N = 97,641)	
Age	76 [70 – 84] (0)	76 [70 – 84] (0)	74 [69 – 81] (0)	74 [69 – 81] (0)	69 [65 – 77]$^{*}$
Sex (Female)	689,078 (56.1%)	76,501 (56.1%)	505,944 (57.6%)	56,137 (57.5%)	813,074 (55.8%)
Socioeconomical status					
*1 (Less deprived)*	137,021 (13.2%)	15,271 (13.2%)	98,412 (13.2%)	10,838 (13.1%)	163,452 (13.2%)
*2*	313,985 (30.2%)	34,845 (30.2%)	224,830 (30.1%)	24,972 (30.1%)	373,120 (30.2%)
*3*	347,455 (33.4%)	38,691 (33.5%)	249,908 (33.5%)	27,686 (33.3%)	411,920 (33.4%)
*4*	197,561 (19.0%)	21,905 (19.0%)	141,999 (19.0%)	16,100 (19.4%)	233,724 (18.9%)
*5 (More deprived)*	43,736 (4.2%)	4,751 (4.1%)	31,295 (4.2%)	3,423 (4.1%)	51,594 (4.2%)
*Missing*	325061	21019	132319	14622	222242
Follow-up time (years)	9 [5 – 10]	9 [5 – 10]	10 [8 – 10]	10 [8 – 10]	8 [4 – 10]
History considered in the model (years)	8 [4 – 9]	8 [4 – 9]	4 [3 – 4]	4 [3 – 4]	–
Outcomes					
All-cause mortality	297,761 (24.2%)	32,886 (24.1%)	171,380 (19.5%)	19,078 (19.5%)	355,901 (24.4%)
Nursing home admission	108,414 (8.8%)	12,172 (8.9%)	81,238 (9.2%)	9,006 (9.2%)	122,844 (8.4%)
Home care need	173,126 (14.1%)	19,357 (14.2%)	131,645 (15.0%)	14,527 (14.9%)	194,052 (13.3%)

*Note:* Individuals with less than 2 and 6 years were eliminated for the development of the 1-year and 5-year models, respectively, for having less history than necessary. Quantitative variables are described as median [Q1 - Q3] and categorical variables as absolute and relative frequencies. Missing values were excluded in the calculation of relative frequencies. $^*$: reports age at cohort inclusion

**Table 3:** Overall performance metrics (mean [95% CI]) on the test set of the different tasks for ARIADNEhr and the baseline models

	All-cause mortality		Nursing home admission		Home care need	
	1 year ahead	5 years ahead	1 year ahead	5 years ahead	1 year ahead	5 years ahead
ARIADNEhr						
Cohen’s Kappa	**0.826 [0.823, 0.830]**	**0.790 [0.785, 0.795]**	0.810 [0.804, 0.815]	**0.424 [0.413, 0.434]**	0.808 [0.803, 0.813]	**0.412 [0.404, 0.420]**
ROC-AUC	**0.905 [0.903, 0.907]**	**0.872 [0.869, 0.875]**	0.869 [0.865, 0.872]	0.685 [0.680, 0.690]	0.865 [0.862, 0.868]	0.693 [0.689, 0.697]
PR-AUC	**0.790 [0.786, 0.795]**	**0.735 [0.729, 0.741]**	0.711 [0.703, 0.719]	0.280 [0.271, 0.290]	0.741 [0.735, 0.747]	0.325 [0.318, 0.332]
Precision	**0.893 [0.890, 0.896]**	**0.902 [0.897, 0.906]**	0.927 [0.922, 0.932]	0.559 [0.546, 0.571]	0.956 [0.953, 0.959]	0.537 [0.527, 0.576]
Recall	**0.842 [0.839, 0.846]**	**0.764 [0.758, 0.771]**	**0.743 [0.735, 0.750]**	**0.403 [0.393, 0.413]**	**0.736 [0.730, 0.742]**	**0.454 [0.447, 0.463]**
XGBoost						
Cohen’s Kappa	0.186 [0.175, 0.201]	0.008 [0, 0.012]	**0.842 [0.829, 0.866]**	0.387 [0.345, 0.419]	0.839 [0.820, 0.845]	0.304 [0.298, 0.309]
ROC-AUC	0.839 [0.834, 0.842]	0.797 [0.792, 0.801]	0.967 [0.943, 0.985]	0.876 [0.862, 0.881]	0.960 [0.951, 0.970]	0.854 [0.849, 0.867]
PR-AUC	0.263 [0.245, 0.276]	0.183 [0.151, 0.190]	0.843 [0.826, 0.867]	0.507 [0.501, 0.512]	0.853 [0.849, 0.858]	0.462 [0.458, 0.465]
Precision	0.685 [0.657, 0.709]	0.548 [0.522, 0.568]	**0.997 [0.981, 1]**	0.899 [0.871, 0.913]	1 [0.988, 1]	0.809 [0.800, 0.819]
Recall	0.111 [0.093, 0.128]	0.004 [0, 0.008]	0.735 [0.704, 0.772]	0.257 [0.255, 0.260]	0.731 [0.729, 0.734]	0.203 [0.189, 0.254]
Random forest						
Cohen’s Kappa	0.119 [0.103, 0.134]	0.002 [0, 0.004]	0.806 [0.796, 0.817]	0.377 [0.363, 0.393]	**0.839 [0.831, 0.847]**	0.314 [0.302, 0.325]
ROC-AUC	0.898 [0.893, 0.904]	0.828 [0.822, 0.834]	**0.978 [0.976, 0.980]**	**0.905 [0.900, 0.909]**	**0.970 [0.968, 0.972]**	**0.885 [0.881, 0.889]**
PR-AUC	0.329 [0.311, 0.345]	0.217 [0.205, 0.229]	**0.853 [0.844, 0.863]**	**0.527 [0.513, 0.541]**	0.854 [0.847, 0.862]	**0.483 [0.471, 0.495]**
Precision	0.782 [0.734, 0.828]	0.714 [0.340, 1]	0.990 [0.986, 0.994]	**0.917 [0.902, 0.932]**	**1 [1, 1]**	0.796 [0.777, 0.813]
Recall	0.066 [0.057, 0.075]	0 [0, 0]	0.687 [0.671, 0.701]	0.248 [0.237, 0.260]	0.735 [0.720, 0.743]	0.212 [0.210, 0.221]
Logistic regression						
Cohen’s Kappa	0.112 [0.098, 0.126]	0.070 [0.060, 0.080]	0.760 [0.749, 0.772]	0.365 [0.351, 0.379]	0.837 [0.829, 0.845]	0.323 [0.310, 0.336]
ROC-AUC	0.887 [0.881, 0.893]	0.836 [0.830, 0.842]	0.961 [0.957, 0.965]	0.901 [0.897, 0.905]	**0.970 [0.968, 0.972]**	**0.885 [0.881, 0.889]**
PR-AUC	0.230 [0.216, 0.244]	0.229 [0.216, 0.241]	0.800 [0.789, 0.812]	0.459 [0.444, 0.474]	**0.856 [0.848, 0.845]**	0.464 [0.450, 0.476]
Precision	0.464 [0.421, 0.510]	0.494 [0.441, 0.549]	0.866 [0.854, 0.878]	0.669 [0.649, 0.689]	0.987 [0.984, 0.990]	0.707 [0.687, 0.726]
Recall	0.067 [0.059, 0.075]	0.041 [0.035, 0.047]	0.688 [0.674, 0.702]	0.269 [0.257, 0.280]	0.735 [0.723, 0.747]	0.230 [0.220, 0.240]

*Note:* The best results for each metric and outcome are highlighted in bold

**Fig. 2:**
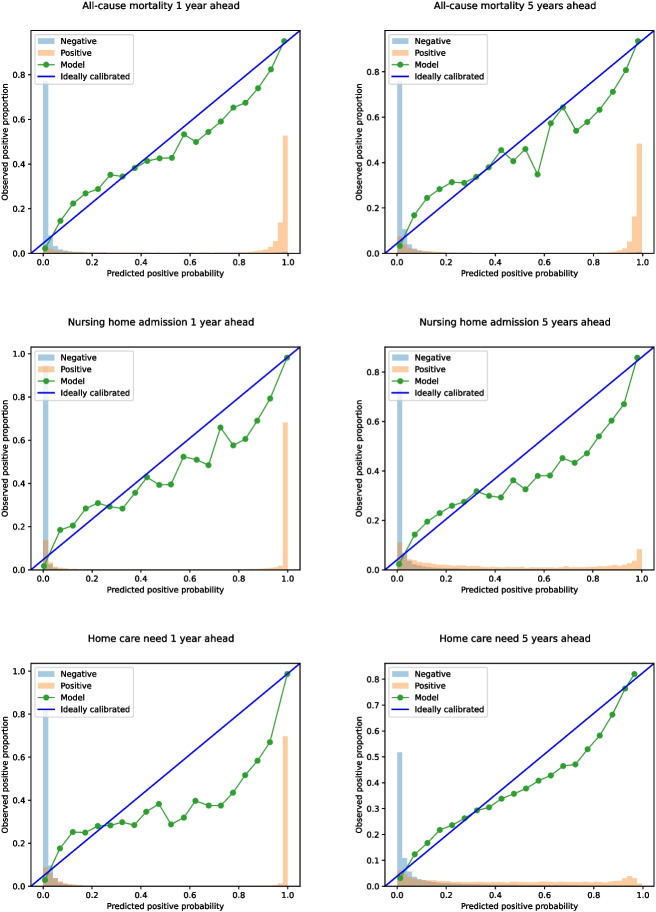
Calibration plots showing the real outcome proportion (y-axis) and the predictive outcome probability (x-axis) from ARIADNEhr. *The teal line corresponds to the calibration curve of the model. For instance, its first point corresponds to the proportion of true positives the model predicted as having a predicted probability of death lower than 0.05 (i.e, 1/20). The blue and orange bars report the real proportion of participants in the negative (i.e., not having the outcome) and positive (i.e., having the outcome) classes, respectively, for each predicted positive probability*

### Model Discriminability and Calibration

Table 3 reports the performance of ARIADNEhR on the test set for each outcome and prediction window, compared to that obtained by baseline methods. For all-cause mortality, ARIADNEhr achieved good overall performance for both 1- and 5-year predictions. The simpler baseline models failed to predict the minority class (i.e., death). For the 1-year prediction of nursing home admission and home care need, the baseline models performed similarly or even slightly better than ARIADNEhr. For the 5-year prediction, ARIADNEhr performed better for both outcomes but without achieving a good enough ROC-AUC.

**Fig. 3:**
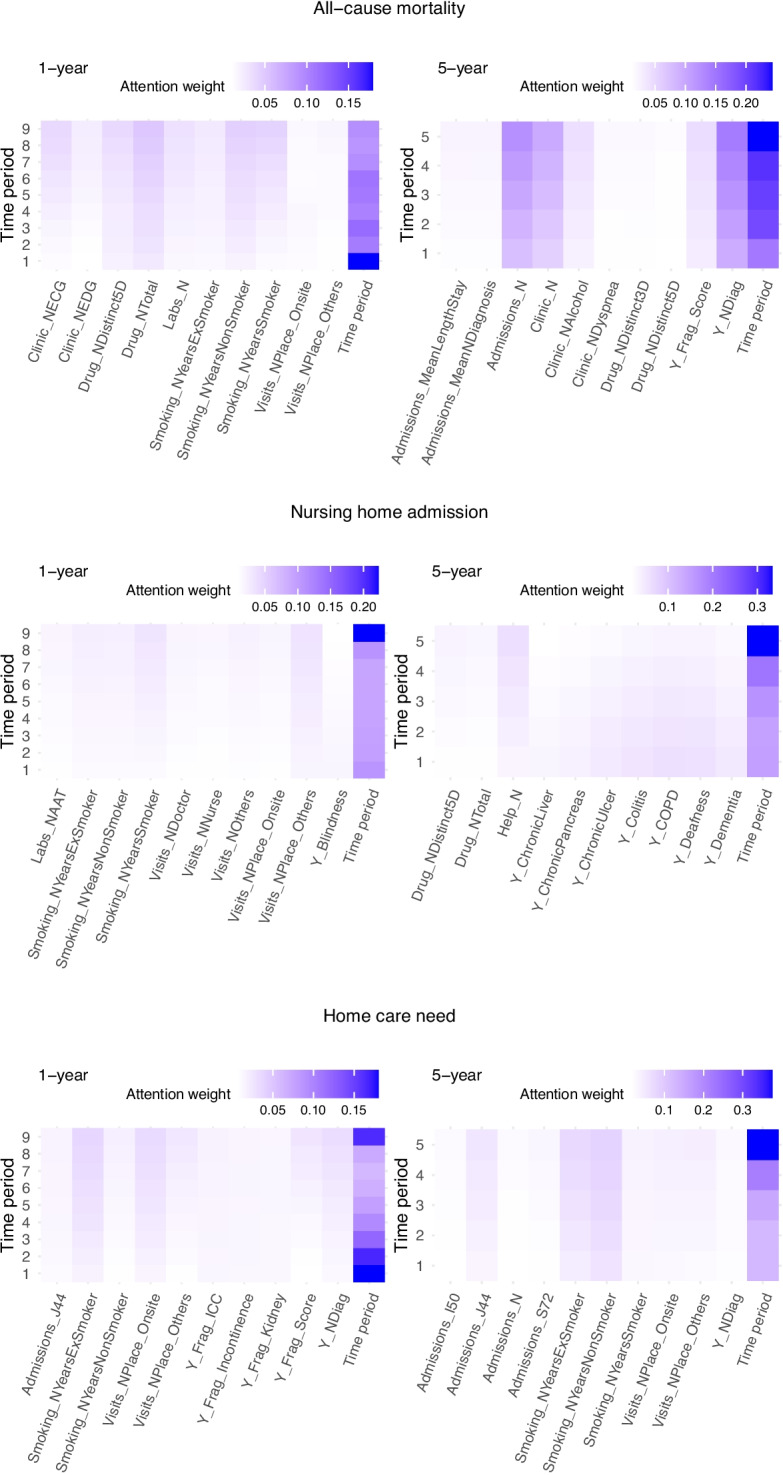
Population level attention maps for each outcome and prediction window. *Note:* These maps were obtained by averaging the attention weights for each individual on the test set and selecting the 10 variables with the highest average contribution. They show the top 10 variables with higher attention weights in the first 10 columns, and the attention weights for the time periods (i.e. years of history; the higher, the closer to the moment when the prediction is performed) in the last column. The description for each variable can be found in [24]

Figure 2 shows the calibration plots and the probability distribution for each task. The one-year prediction models showed a good separation of probability distributions, although the nursing home and home care models showed a higher number of false negatives. In the case of the five-year models, it was still good only for the case of all-cause mortality, as the probability of positive predictions for nursing home and home care need presented a much more heterogeneous distribution.

In addition to the overall performance, two stratified analyses of ARIADNEhr’s performance were conducted. As Fig. S4 shows, the performance was higher in women, being higher in outcomes related to utilization of specialised care resources than in mortality. ARIADNEhr performed better with people who started the study at a higher age (see Fig. S5). The difference in performance between age groups was larger when predicting resource utilization with a larger prediction window (i.e. 5-year vs 1-year).

**Fig. 4:**
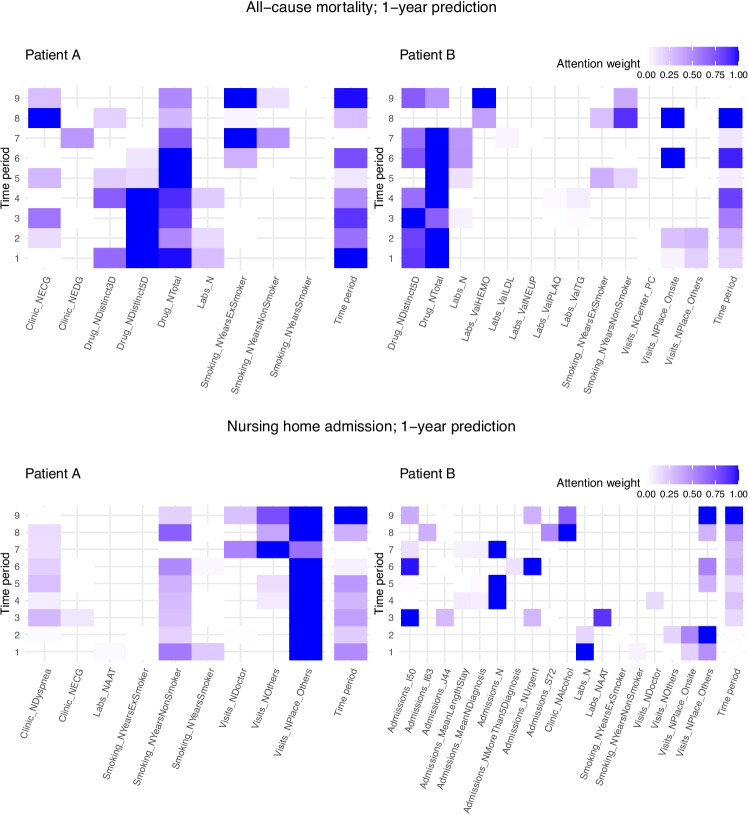
Attention maps for two participants, and two 1-year outcomes. *Note:* For each year, the 5 most contributing characteristics are shown in color, which need not be the same every year. The attention weights have been normalized between 0 and 1 considering only the values shown, just to improve the visualization. The description for each variable can be found in [24]

### Model Transparency and Attention Maps

Figure 3 shows the top 10 contributing features according to the population-level attention maps, and the time-level attention weights, for each outcome and prediction window. It should be highlighted that the variable-level attention weights are substantially lower than the time-level due to the large number of variables that were being considered simultaneously.

The closest time period to the time of the prediction was the most contributing, except for 1-year mortality prediction, in which the information on the initial status of each participant seemed to be more determinant. The variables with the greatest overall contribution to 1-year all-cause mortality were in most cases proxies of frailty (i.e. falls) and critical conditions (such as heart disease, which is monitored with ECGs). Information related to visits to primary care centres contributed the most to nursing home admission at one year, while events related to limiting conditions (e.g. COPD, femur fracture, congestive heart failure) contributed the most to home care need.

Figure 4 shows four patient-level attention maps, which would be closer to the actual use of attention maps in the clinical practice. It shows the attention maps for 1-year all-cause mortality and nursing home admission prediction for patients A (sex: female, age when prediction: 86, socioecomic index: 2), and B (sex: male, age when prediction: 95, socioecomic index: 3). Patient A neither died nor was admitted to nursing home during the following year, while patient B had both outcomes. In these patients, the most contributing time periods for determining nursing home admission were the later ones, whereas their contribution is more distributed throughout the patient’s history for predicting all-cause mortality. Regarding all-cause mortality, the variables related to the amount of drugs consumed and years of smoking were highly determinant for both patients. On the other hand, in the patient who died, some importance was given also to other variables such as the number of visits to primary care and the haemoglobin value in the last year. Regarding the nursing home admission, in person attendance of primary care consultations was very important for the patient who was not admitted to a nursing home, while other information related to frailty and the need for special care, such as hospital admissions, was contributing for the patient who did.

## Discussion

In this study, we developed ARIADNEhr, a prediction model tailored to handle EHR intricacies (e.g. missing data, sparsity, and heterogeneity, etc.) to predict 1-year and 5-year all-cause mortality, nursing home admission, and home care need in the older population. We compared its performance against diverse baseline machine learning algorithms and between population groups. ARIADNEhr incorporates two-level attention mechanisms to enhance transparency and illustrate which clinical variables and time periods were the most determinant to the outcome.

ARIADNEhr outperformed baseline methods when predicting 1- and 5-year all- cause mortality achieving Cohen’s Kappa (which accounts for class imbalance) 3.4 and 10.3 times higher, respectively. For 1-year nursing home admission or home care need, ARIADNEhr matched or slightly outperformed baseline methods. Predicting these two outcomes at 5 years yielded unsatisfactory results across all methods. Overall, ARIADNEhr performed better in women, likely due to their higher representation in older populations. Precision was higher in men for all-cause mortality and higher in women for the other two outcomes, possibly reflecting the higher prevalence of each outcome in each sex [26]. Performance improved with patient age, consistent with the increased frequency of these outcomes with age [26].

Prior studies have developed prediction models for these outcomes, but varying study designs and limitations make direct comparisons challenging. In [27], 1-year to 10-year all-cause mortality was predicted using Gradient Boosted Trees and 20 cross-sectional EHR variables in the general population, achieving ROC-AUCs between 0.85 to 0.88 for 1-year all-cause mortality and 0.80 to 0.83 for 5-year all-cause mortality. Krasowski et al. predicted up to 5-year all-cause mortality in individuals aged 75 years or older using aggregated versions of up to 5 years of claims history and different machine learning algorithms, reporting balanced accuracies between 0.65 and 0.67 [28]. In [29], predicted 1-year nursing home placement within home- and community-based services participants using cross-sectional data and achieved an ROC-AUC of 0.76, 12.5% lower than ARIADNEhr’s. A systematic review of studies predicting the need for supportive services (home care or nursing home included) identified models using cross-sectional data with ROC-AUC ranging 0.70-0.80 [30]. A systematic review of models using longitudinal EHR data found 16 studies predicting mortality, reporting ROC-AUC between 0.78 and 0.98 but none was found on specialized care services utilization [31]. These types of outcomes deserve more attention from the research community using more complex models.

Our study has some strengths. First, we used a large and representative real-world EHR dataset, both in terms of participants and variables. Second, we compared the proposed model against models using cross-sectional data. Third, the performance achieved by most models is high considering class imbalance. Finally, by incorporating two-level attention mechanisms, transparency and clinical utility are enhanced both at the population and the individual level. However, these results should be interpreted in light of some limitations. First, ending up using home care or a nursing home might depend on the system’s capacity and not only on the person’s needs, so there may be mislabeled individuals. While attention maps can be useful in increasing model transparency [32, 33], their role in increasing interpretability may be limited, according to recent discussions [33–35].

Future work should consider externally validating these models both geographically (i.e., with a different population) and temporally (i.e., same population but in a different time period), or incorporating new data types (e.g., free text, which could be extraordinarily informative in primary care). Other data sources such as private health insurance, enviromental data or economic indicators could also be included. ARIADNEhr could also be tested for predicting different outcomes and different prediction windows.

In sum, we present a novel, transparent, deep learning architecture that can rely on real-world EHR data for predicting three health-related outcomes of interest in the older population. The performance of the resulting models, especially those of all-cause mortality and 1-year healthcare resource utilisation, could prove valuable for further efforts in implementation in clinical practice to guide resource planning or patient treatments. In addition, the variables suggested in this paper as contributing most to outcomes can be further studied as risk factors.

## Supplementary Information

Below is the link to the electronic supplementary material.

Supplementary file 1 (pdf 567 KB)

Supplementary file 2 (docx 91 KB)

## Data Availability

The data used in this study are confidential and are only available for the participating researchers, in accordance with current European and national laws. Thus, the distribution of the data is not allowed. However, researchers from public institutions can request data from SIDIAP. Further information is available online (https://www.sidiap.org/index.php/en/solicituds-en).

## Code Availability

The code to define, train, and internally validate the model can be found in GitHub [24]. This GitHub also provides some mock data to be able to run the scripts.
