# Supplementary material for: Development of Attention-based Prediction Models for All-cause Mortality, Home Care Need, and Nursing Home Admission in Ageing Adults in Spain Using Longitudinal Electronic Health Record Data
Source: J Med Syst. 2025 Jan 25;49(1):17. doi: 10.1007/s10916-024-02138-z (PMC11762432; doi:10.1007/s10916-024-02138-z)
Supplement: Supplementary file 1 — (pdf 567 KB) [file 10916_2024_2138_MOESM1_ESM.pdf]

Supplementary Material

This document includes the supplementary material and figures of the manuscript **"Development of attention-based prediction models for all-cause mortality, home care need, and nursing home admission in ageing adults in Spain using longitudinal electronic health record data"**, authored by Lucía A. Carrasco-Ribelles, Margarita Cabrera-Bean, Sara Khalid, Albert Roso-Llorach, and Concepción Violán.

Table of Content

Study population **1**

ARIADNEhr **2**

Data representation 2
Data input and preprocessing 2
Attention at variable level 2
Attention at time level 4
Concatenate static variables and outcome prediction 4

Training details **5**

Supplementary figures **5**

Study population

The flow chart of the study population is shown in Figure S1.

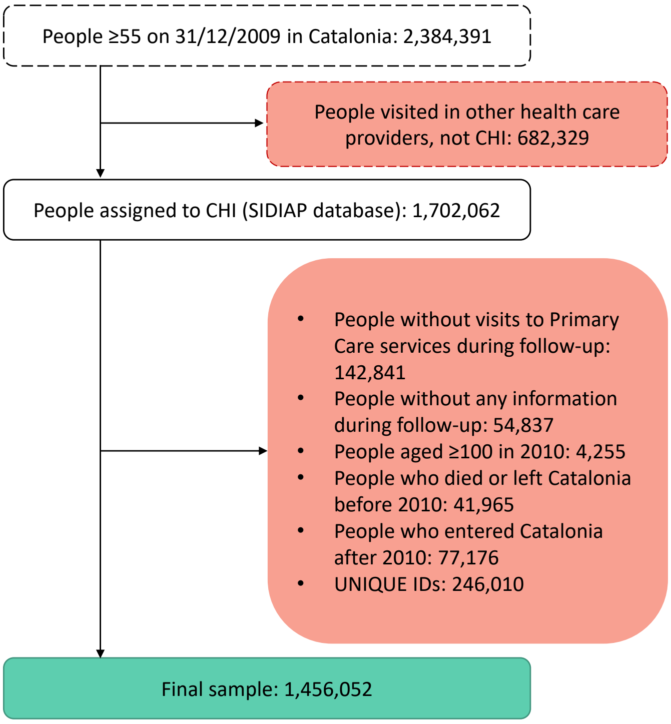

Fig. S1: Flow chart of the study population.
Note: The figure reports the number of individuals who met exclusion criteria, as well as the number of individuals that met all the criteria (unique IDs).

ARIADNEhr

In this section, we first introduce the notations and then present the proposed architecture for obtaining predictions from longitudinal EHRs, which consisted of an input and preprocessing block, followed by a time-distributed block to obtain the attention maps at the feature level, then a block to obtain the attention map at the time period level, and finally a prediction block, as shown in Figue S2.

Data representation

Given a set of N subjects, each n subject was defined by a set of K and S variables. K variables, which could be either numeric (K_{num}) or categorical (K_{cat}), i.e. $K = K_{num} + K_{cat}$, were measured over time, constituting time series of length T_n, where T_n is the number of consecutive years of recorded EHR for each n subject, so temporal length of input variables can differ among individuals. Each subject n was then defined by a matrix of time series $\mathbf{X_d^n} = [\mathbf{x_1^n}, ..., \mathbf{x_j^n}, ..., \mathbf{x_K^n}] \in \mathbb{R}^{T_n \times K}$, with $\mathbf{x_j^n}$ representing the time series of the j-th variable and length T_n, and $x_{t,j}^n$ the value of the j-th variable in time t. On the other hand, S variables were static, such as sex assigned at birth. Each subject n was then also defined by a vector $\mathbf{x_s^n} = [x_1^n, .., x_j^n .., x_S^n] \in \mathbb{R}^S$. To handle the missing values by applying masking, $\mathbf{m_s^n}$ and $\mathbf{M_d^n}$ were defined as a vector and a matrix, respectively, of equal dimension to $\mathbf{x_s^n}$ and $\mathbf{X_d^n}$ to indicate missingness. Specifically, $m_{p_j}^n = 1$ if x_j^n was present, otherwise $m_{p_j}^n = 0$ and x_j^n was set to -100. Similarly, $m_{d_{t,j}}^n = 1$ if $x_{t,j}^n$ was present, otherwise $m_{d_{t,j}}^n = 0$ and $x_{t,j}^n$ was set to -100. When considering the complete set of subjects, $\mathbf{X_d} \in \mathbb{R}^{N \times T_n \times K}$ (Figure S3A), and $\mathbf{x_s} \in \mathbb{R}^{N \times S}$ (Figure S3B) represented the data, and $\mathbf{M_d} \in \mathbb{R}^{N \times T_n \times K}$ and $\mathbf{m_s} \in \mathbb{R}^{N \times S}$ the missingness masking matrices.

Data input and preprocessing

First, categorical variables were one-hot-encoded, increasing the dimension K_{cat} accordingly. Then, to make T_n consistent among subjects, every $\mathbf{X}_d^n$ and $\mathbf{M}_d^n$ were pre-zero-padded (i.e. right-alignment [1, 2]) up to $T = max(T_1, ..., T_n)$, to address different starting or ending times of follow-up (Figure S3C and Figure 1A-B). To reduce the differences in the amount of zero-padding between subjects, we first performed a temporal aggregation of the observations, calculating the mean, mode, or count of the observations in each time period according to the type of data. An additional masking matrix per subject, $\mathbf{L^n} \in \mathbb{R}^{T \times K}$, was defined to indicate this completion. Specifically, $l_{t_j}^n = 1$ for every $t \leq T_n \leq T$ and $l_{t_j}^n = 0$ for every $t > T_n$. When $l_{t_j}^n = 0$, $x_{t,j}^n \ \forall \ 1 \leq j \leq K$ was set to -99. Then, masking was single for S static variables and double for K dynamic variables, i.e. missing values and temporal zero-padding.

After masking, K_{num} numeric variables were normalized, i.e. shifted and scaled into a distribution centred around zero and standard deviation 1, and then concatenated to K_{cat} categorical variables. The overall architecture can be seen in Figure S2. Hereafter, for the sake of simplifying notation, the superscript n, which represents the n-th subject, is omitted.

Attention at variable level

Next, the attention of each one of the dynamic K variables, $x_{t,j}$, was calculated for each time point $t \ \forall \ 1 \leq t \leq T$, where the resulting attention weights ($\boldsymbol{\alpha}_{\mathbf{V_t}}$) represent the contribution of each $x_{t,j}$ to the outcome prediction. For this purpose, T blocks formed by a recurrent layer (RL) with U neurons, followed by the attention layer described in [3] was created and then used to process every input vector $\mathbf{x}_t = [x_{t,1}, ..., x_{t,K}]$, as shown below:

for t *in* $1 : T$ **do**
  $\mathbf{H_t} := [\mathbf{h}_{t,1}, ..., \mathbf{h}_{t,j}, ..., \mathbf{h}_{t,K}] = RL_t(x_{t,1}, ..., x_{t,j}, ..., x_{t,K})$
  $\boldsymbol{\alpha}_{\mathbf{V_t}} = softmax({\omega_{\mathbf{1_t}}}^\top tanh(\mathbf{H_t}))$
  $\mathbf{h_t^*} = tanh(\mathbf{H_t} \boldsymbol{\alpha}_{\mathbf{V_t}})$
end for

where $(.)^\top$ denotes transpose, $\mathbf{H_t} \in \mathbb{R}^{U \times K}$, $\omega_{1_t} \in \mathbb{R}^U$ represents a trained parameter vector, $\boldsymbol{\alpha}_{\mathbf{V_t}} \in \mathbb{R}^K$ is the vector of attention weights of variables $x_{t,j} \ \forall 1 \leq j \leq K$ for each t, and $\mathbf{h_t^*} \in \mathbb{R}^U$ the representation of the original variables for each k after the recurrent layer. After every x_t is processed, each $\boldsymbol{\alpha}_{\mathbf{V_t}}$ is concatenated to form $\boldsymbol{\alpha}_{\boldsymbol{V}} := [\alpha_{\mathbf{V_1}}, ...\alpha_{\mathbf{V_t}}, ..., \alpha_{\mathbf{V_T}}]^\top \in \mathbb{R}^{T \times K}$. Matrix $\boldsymbol{\alpha}_{\boldsymbol{V}}$ contains the contribution of each variable in each time period to the outcome prediction.

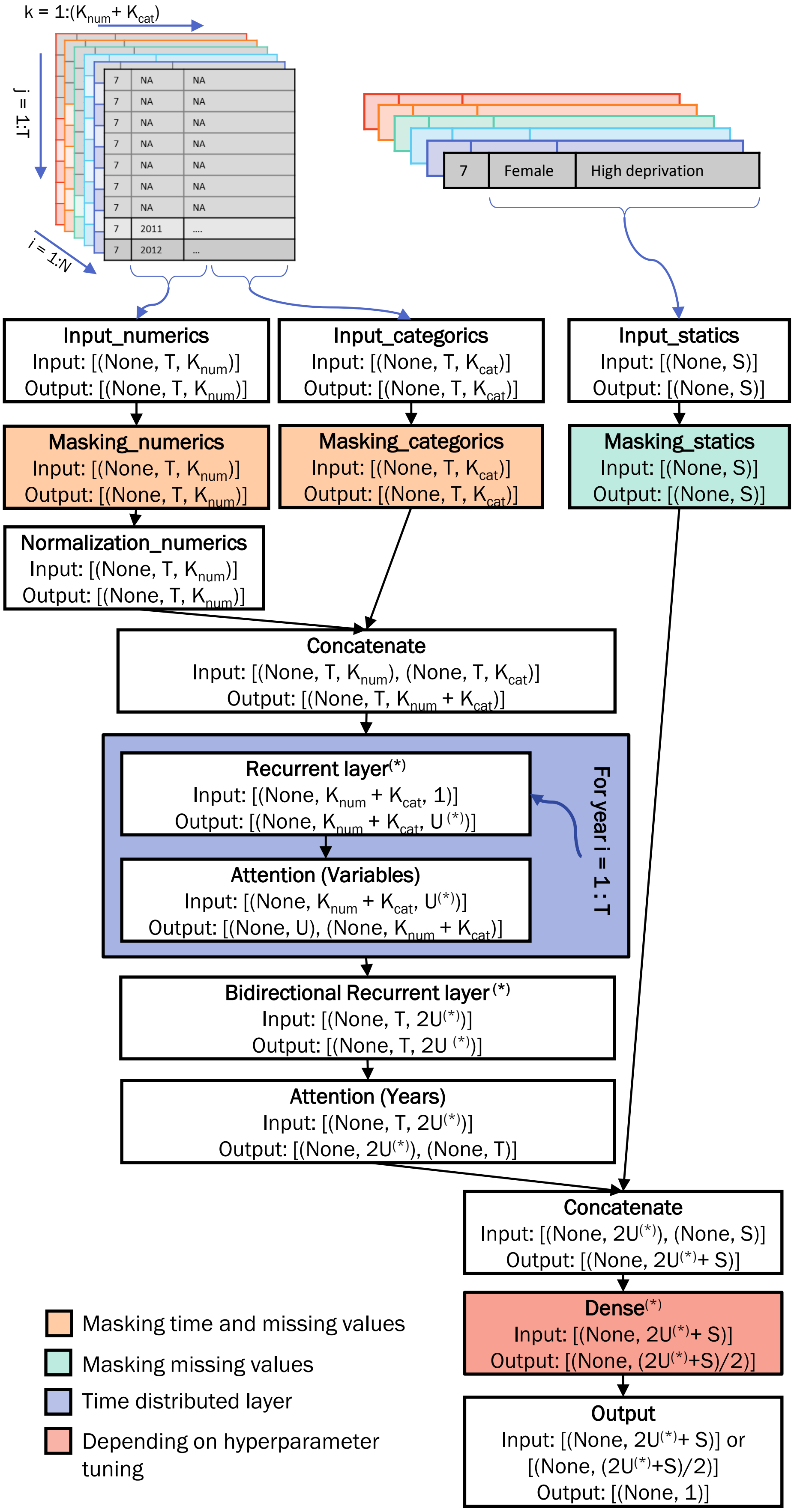

Fig. S2: ARIADNEhr architecture.
None represents the dimension of the individuals (N), and its value depends on the batch size. $^{(*)}$ The type of recurrent layer (GRU or LSTM), the number of units U (64, 128, 256, or 512), and the presence of a Dense layer before the output were determined through hyperparameter tuning.

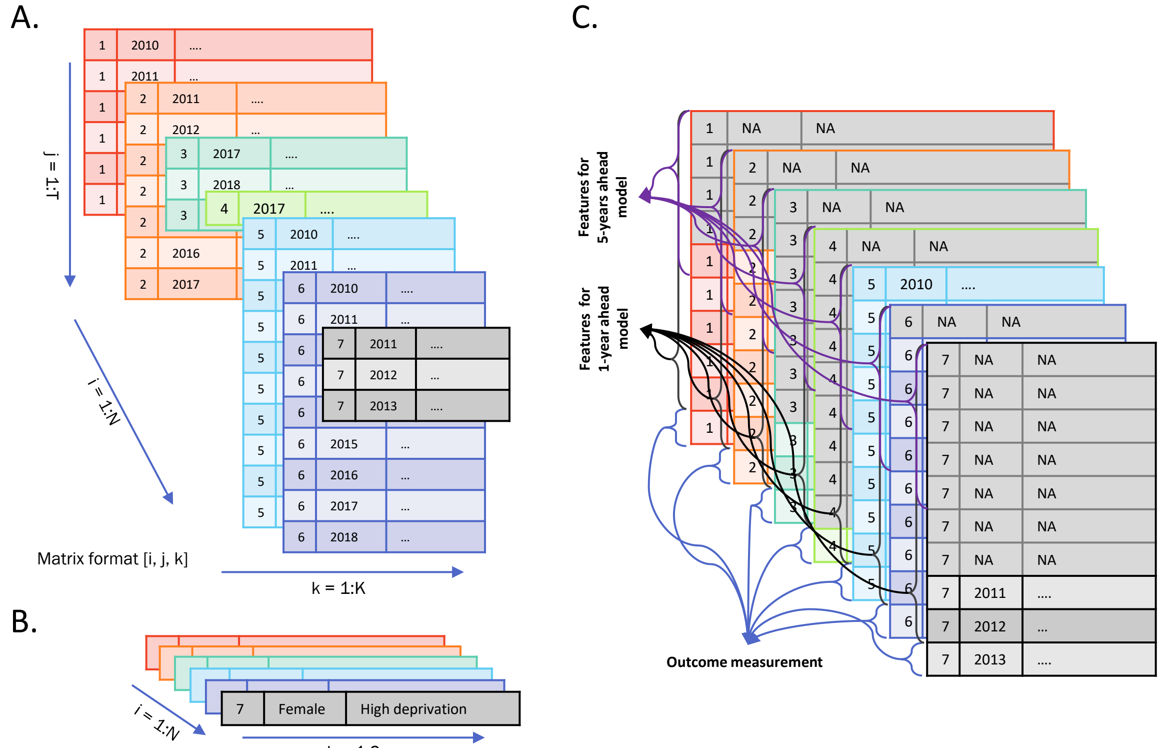

Fig. S3: From the original source data to the input data matrix.
Figure S3A shows how the original dynamic source data are arranged into a matrix per subject, after annually aggregating the available information, forming a tensor. Figure S3B shows how the original static source data are arranged into a vector by subject, forming a matrix. Figure S3C shows the tensor from Figure S3A after zero padding to make its j dimension consistent for every patient. It highlights which features are considered in each model, according to the prediction window.

Attention at time level

To obtain the attention weights of each time period according to their contribution to the outcome, we used the same approach as in the previous step and created a block formed by a bidirectional recurrent layer (BiRL), followed by the attention layer described in [3]. Given $\mathbf{h_1^*}, ..., \mathbf{h_1^*}, ..., \mathbf{h_T^*} \in \mathbb{R}^U$:

$$\mathbf{G} := [\mathbf{g_1}, ..., \mathbf{g_j}, ..., \mathbf{g_T}] = BiRL(\mathbf{h_1^*}, ..., \mathbf{h_1^*}, ..., \mathbf{h_T^*})$$
$$\boldsymbol{\alpha_T} = softmax({\omega_2}^\top tanh(\mathbf{G}))$$
$$\mathbf{g}^* = tanh(\mathbf{G}\boldsymbol{\alpha_T})$$

where matrix $\mathbf{G} \in \mathbb{R}^{2U \times T}$ $\omega_2 \in \mathbb{R}^{2U}$ represents a trained parameter vector, $\boldsymbol{\alpha_T} \in \mathbb{R}^T$ contains the attention weights of each time period t, and $\mathbf{g}^* \in \mathbb{R}^{2U}$ is the output vector given by the attention layer.

Concatenate static variables and outcome prediction

The obtained vector was then concatenated with the patient's characteristics $\mathbf{x_s} \in \mathbb{R}^S$, achieving a final representation $\mathbf{p}$ of each patient combining the dynamic and static information ($\mathbf{p} = [\mathbf{g}^*, \mathbf{x_s}] \in \mathbb{R}^{2U+S}$). Given this vector $\mathbf{p}$, a softmax layer was incorporated to obtain the outcome prediction. An additional dense, fully connected, layer could be added before the softmax layer is determined by the hyperparameter tuning.

To train the model, a combination of the binary cross-entropy (CE) and the dice coefficient (DC) loss was used as loss function, to strengthen the architecture against the unbalanced outcome

distribution [4]:

$$L = 0.5L_{CE} + 0.5L_{DC}$$

$$L_{CE} = -\frac{1}{N}\sum_{i=1}^{N}[y_i \cdot \log(p_i) + (1 - y_i) \cdot \log(1 - p_i)]$$

$$L_{DC} = 1 - \frac{2 \cdot \sum_i^N p_i \cdot y_i + \epsilon}{\sum_i^N p_i^2 + \sum_i^N y_i^2 + \epsilon}$$

where y_i represents the true label for each sample, p_i represents the predicted binary output, ϵ is a small constant added to avoid division by zero, and N is the total number of subjects.

Training details

Early stopping was defined to prevent overfitting. Training stopped if ROC-AUC did not increase more than 0.001 after 10 epochs, restoring the best weights. In addition, the learning rate was reduced by 0.1 if the ROC-AUC did not change after 5 epochs. Batch size was set to 32, and the maximum number of epochs was 50. The optimizer was Adam, with an initial learning rate of 0.001.

Supplementary figures

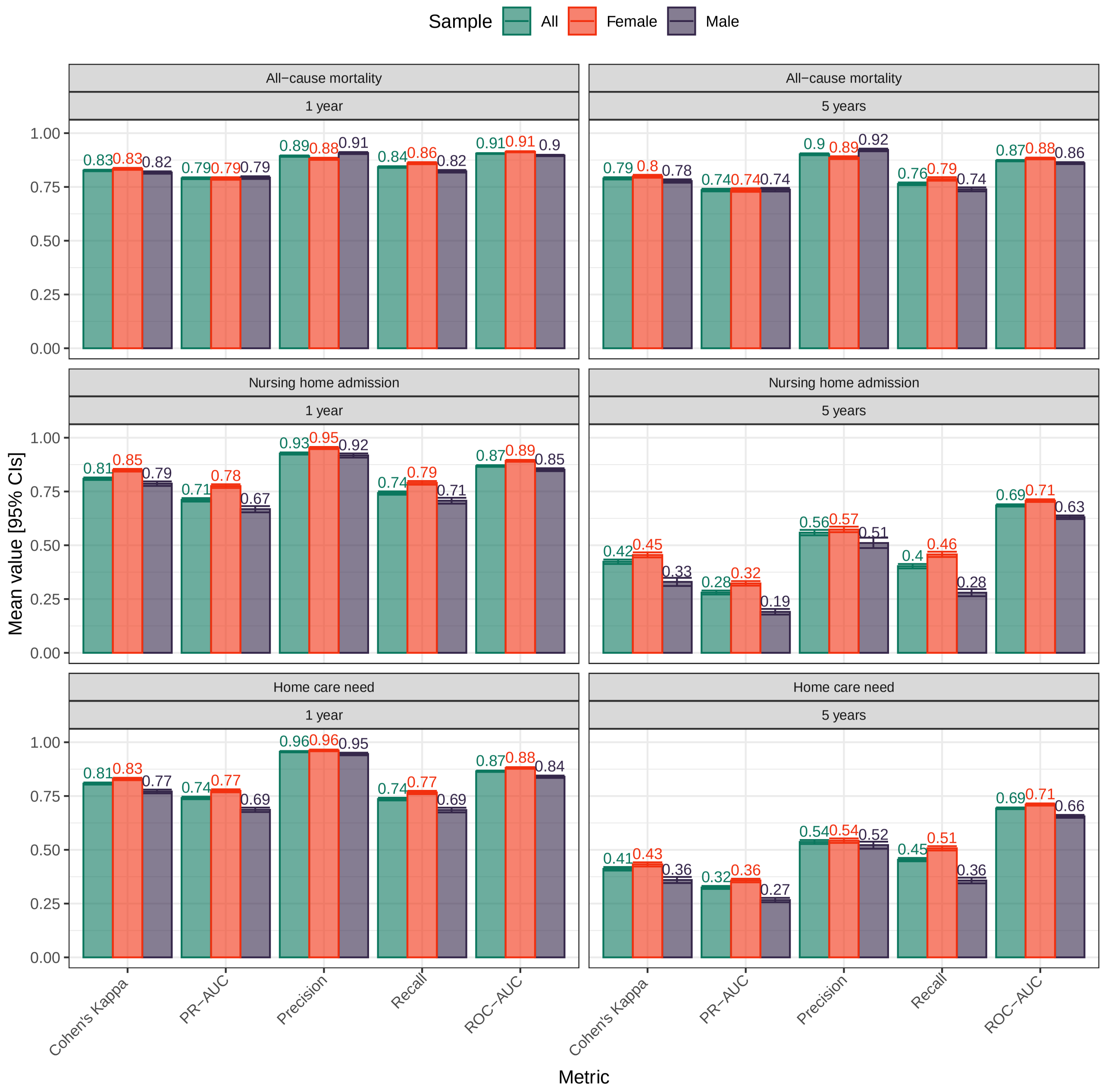

Fig. S4: Performance metrics of ARIADNEhr on the test set, by sex.

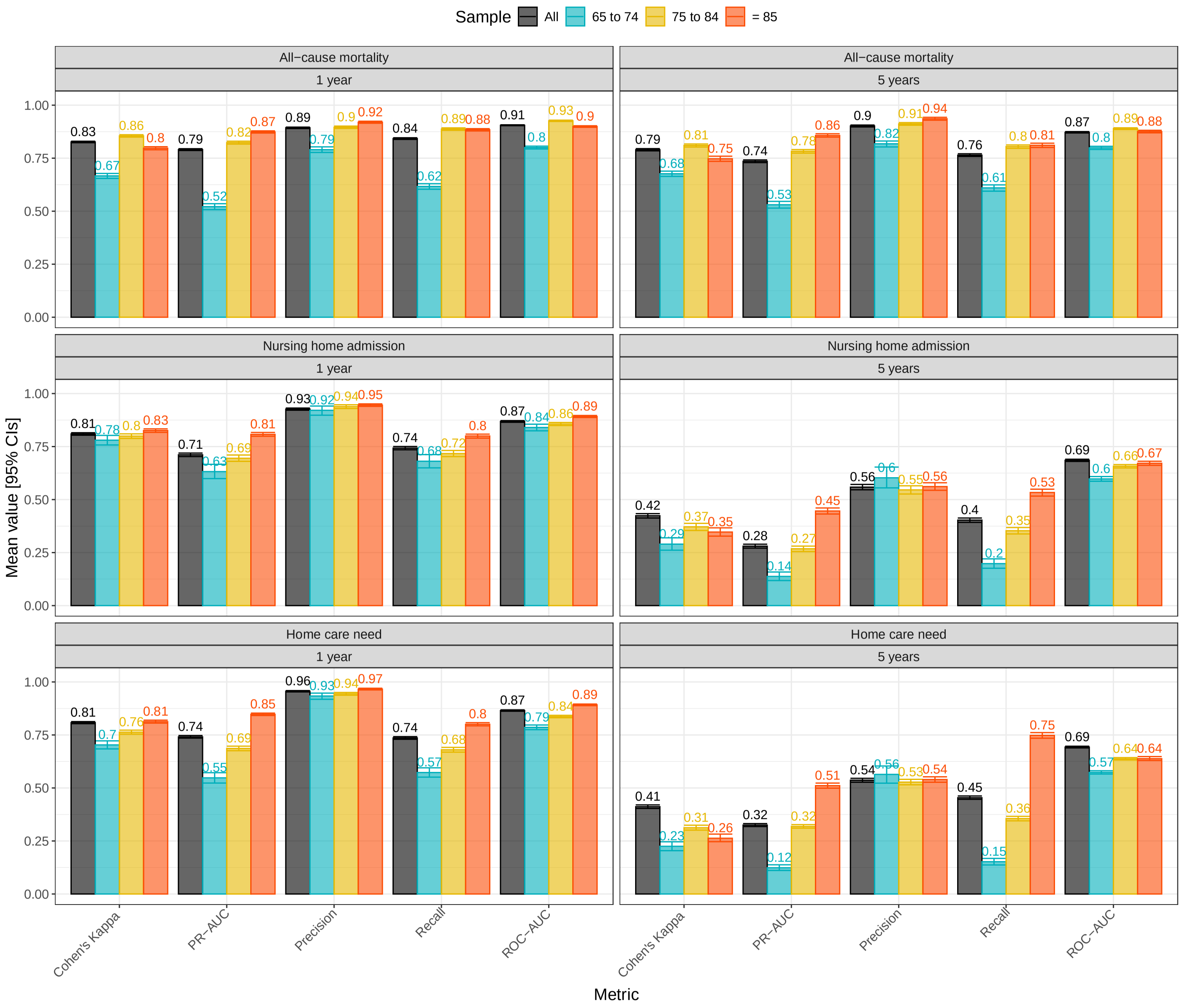

Fig. S5: Performance metrics of ARIADNEhr on the test set, by initial age.

Supplementary Material References

[1] Dwarampudi, M., Reddy, N.V.S.: Effects of padding on LSTMs and CNNs (2019). https://doi.org/10.48550/arXiv.1903.07288

[2] Lopez-del-Rio, A., Martin, M., Perera-Lluna, A., Saidi, R.: Effect of sequence padding on the performance of deep learning models in archaeal protein functional prediction. Scientific Reports **10**(1) (2020) https://doi.org/10.1038/s41598-020-71450-8

[3] Zhou, P., Shi, W., Tian, J., Qi, Z., Li, B., Hao, H., Xu, B.: Attention-based bidirectional long short-term memory networks for relation classification. In: Proceedings of the 54th Annual Meeting of the Association for Computational Linguistics (Volume 2: Short Papers), pp. 207–212. Association for Computational Linguistics, Berlin, Germany (2016). https://doi.org/10.18653/v1/P16-2034 . https://aclanthology.org/P16-2034

[4] Milletari, F., Navab, N., Ahmadi, S.-A.: V-Net: Fully Convolutional Neural Networks for Volumetric Medical Image Segmentation (2016). https://doi.org/10.48550/arXiv.1606.04797
